# Supplementary material for: Neuronal Bmal1 regulates retinal angiogenesis and neovascularization in mice
Source: Commun Biol. 2022 Aug 6;5:792. doi: 10.1038/s42003-022-03774-2 (PMC9357084; doi:10.1038/s42003-022-03774-2)
Supplement: Supplementary file 2 — Supplementary Material [file 42003_2022_3774_MOESM2_ESM.pdf]

Supplementary Figure 1:

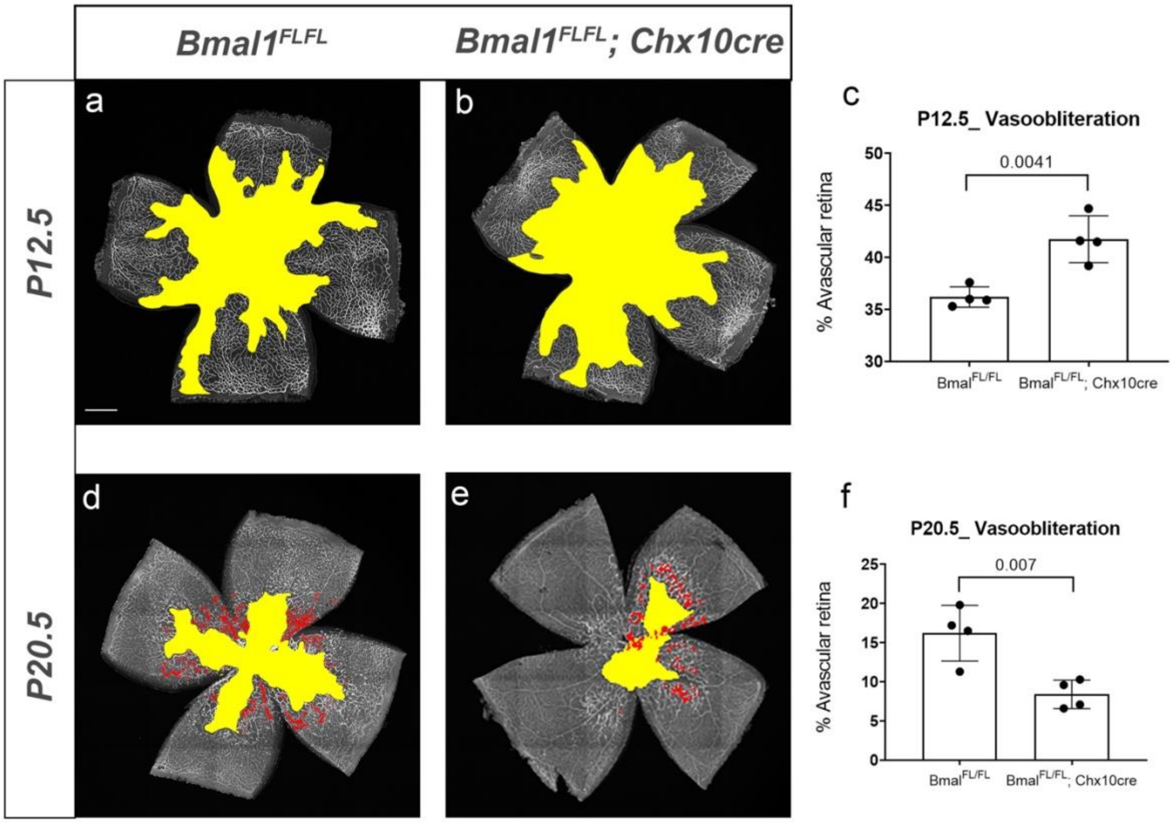

Supplementary Figure 1: **Bmal1 negatively regulates revascularization:** (a-e) Representative images of the P12.5 retinas with avascular area outlined in yellow and neovascular tufts (red) in the control Bmal1$^{FL/FL}$ (a, d), and Bmal1$^{FL/FL}$;Chx10cre (b, e) animals at the indicated ages. (c) Graph showing a significant difference in capillary regression in the mutants compared to the controls, quantified as avascular area at P12.5. (f) Quantitation of the avascular area at P20.5, shows that vessel regrowth is accelerated in the Bmal1$^{FL/FL}$; Chx10Cre animals compared to the controls. n=4, each dot represents average from two eyes. Error bars represent SEM. Scale bar=500µm.

Supplementary Figure 2:

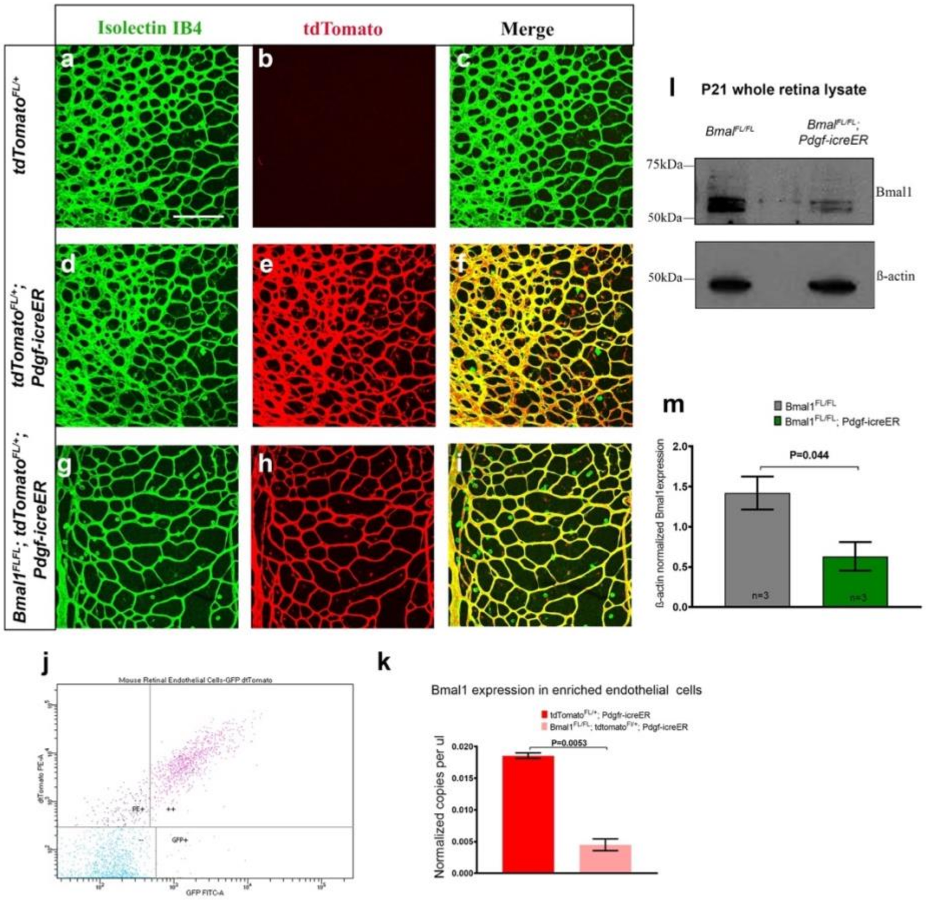

Supplementary Figure 2: **Validation of loss of Bmal1:** Tamoxifen was administered intraperitoneally for three consecutive days starting at day of birth, to elicit cre mediated deletion of Bmal1. (a-i) Retinal vasculature at P7.5 labeled with isolectin (green) and the cre reporter transgene, tdtomato (red) to show specificity of Cre expression in the endothelial cells. (l) Western blot image of retinal lysates probed with Bmal1 and β-actin (housekeeping protein) antibody at P21. (m) Quantification of Bmal1 protein normalized to β-actin, show reduced Bmal1 protein in mutant (Bmal1$^{FL/FL}$; Pdgf-icreER) compared to controls (Bmal1$^{FL/FL}$). (j) Representative fluorescence-activated cell sorting (FACS) plot of retinal endothelial cells to show the selection of the endothelial cell population, identified as tdTomato and GFP double positive. (k) ddPCR quantification of Bmal1 copy numbers in the enriched endothelial cell population, show a significant reduction of Bmal1 copies in the mutant (Bmal1$^{FL/FL}$; tdTomato$^{FL/+}$; Pdgf-icreER) as compared to the control (tdTomato$^{FL/+}$;Pdgf-icreER). n=3. Error bars are represented by SEM. Scale bar=50µm.

Supplementary Figure 3:

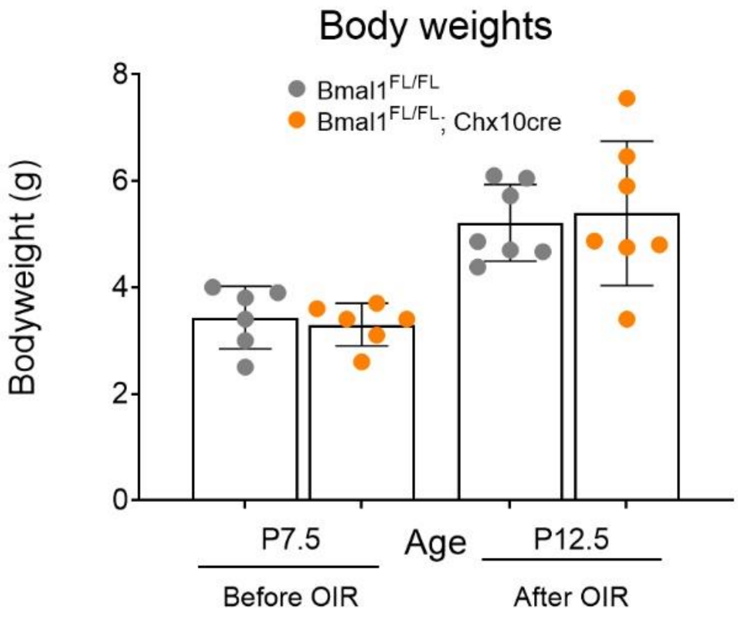

Supplementary Figure 3: **Body weights before and after OIR exposure:** Graph indicates that there is no difference in animal weight between the control (Bmal1$^{FL/FL}$) and mutant (Bmal1$^{FL/FL}$; Chx10cre) before (P7.5) and after (P12.5) the mice were exposed to OIR conditions. n=5-7. Error bars are represented by SEM.

Supplementary Figure 4:

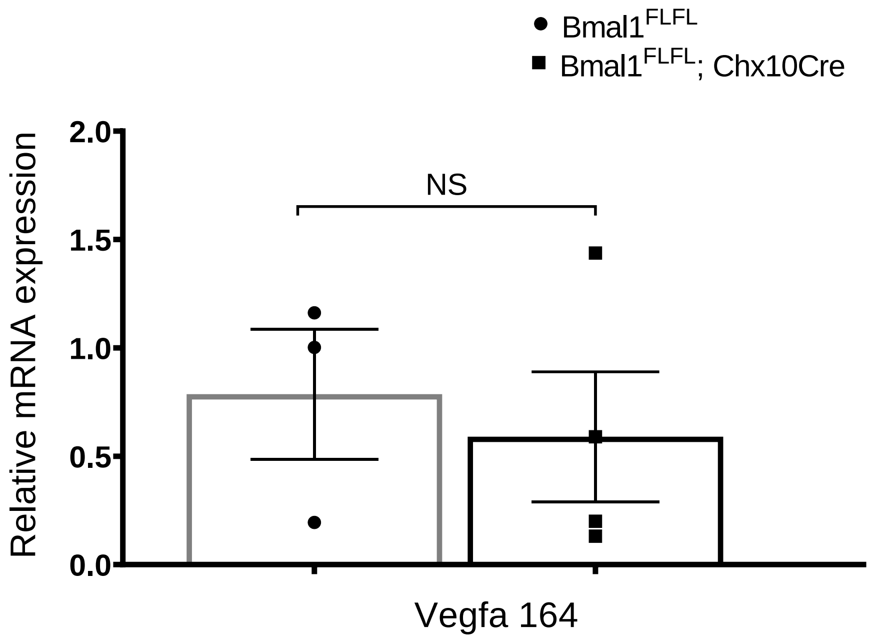

Supplementary Figure 4: **Bmal1 deletion from retinal progenitor cells does not affect Vegfa transcript levels:** Relative retinal mRNA expression of Vegfa 164 in P2.5 (P3) control (Bmal1$^{FL/FL}$) and mutant (Bmal1$^{FL/FL}$; Chx10cre) mice. n=3-4, each dot represents average from two eyes Error bars are represented by SEM.

Supplementary Figure 5

Uncropped western blot probed using Sema6D antibody.

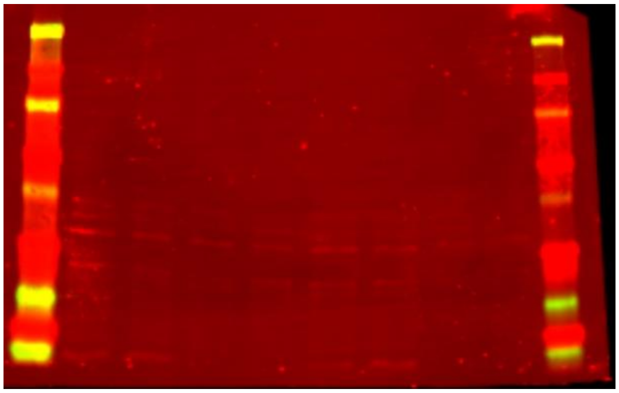

Supplementary Figure 6

Uncropped western blot probed using beta-actin antibody.

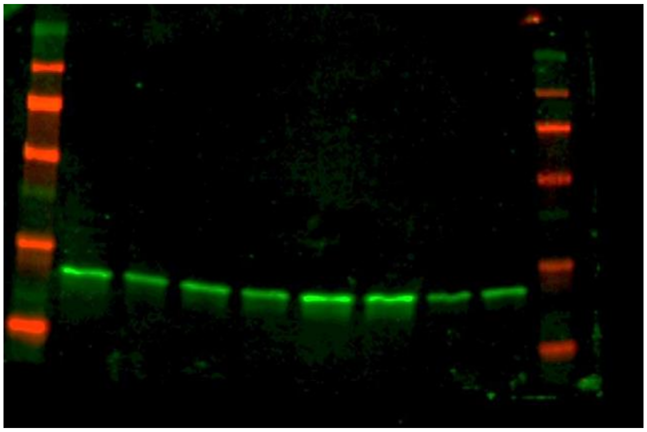

Supplementary Table 1: qPCR primers list.

Gene	Forward	Reverse
Bmal1 Exon 8	GGGCCACAGTCAGATTGAAA	GCTGAACAGCCATCCTTAGC
Sema3A	TGGGATTGCCTGTCTTTT	GGCCAAGCCATTAAAAGTGA
Sema3D	GTTGCTAGCAGGAAGGGTGA	ACAGGAAGAAAGAGCACCGT
Sema6D	CTCTGAAGCTGGCGTGGTACTT	TGAGACCACCTTTCTGTCCTCC
Vegfa 164	AACGATGAAGCCCTGGAGTG	CAAGGCTCACAGTGATTTTCTGG
Actin	TTCTTTGCAGCTCCTTCGTT	ATGGAGGGGAATACAGCCC

Supplementary Table 2:
P3-List of genes with Bmal1 Ebox regions

Aass	Hamp
Asxl2	Haus6
Atp6v0a2	Hmx1
Gm10415	Hottip
Lrrc4c	Igbp1b
Nipbl	Isca1
Otx2os1	Magi2
Parva	Pde10a
Rnf144b	Pdzrn4
Ssh2	Phc1
Strn	Pmvk
Ttll1	Prdm13
Tulp3	Prlr
Unc13c	Sec14l1
Vmn2r78	Sema3A
Ankrd12	Sema3D
Atp2b1	Sema6d
Celf2	Slc44a1
Col13a1	Tmem117
Cyp2u1	Ypel5
Dlg2	Zbtb7b
Eif5	Acaca
Fam13b	Dsg1b
Fgd1	Enox2
Fstl1	Gm16675
Galnt13	Gm31517
Gfra2	Kirrel3
Gm20754	Ttc6
Gnaq	4930529I22Rik
Gm25339	Aatf
Gm42860	Cyp2b26-ps
Gm48779	Dsg1a
Gm49634	Eif4b
Irf2	Gm12684
Kirrel3os	Gm12692
Lcmt1	Gm13498
Man2a1	Gm13678
Nek10	Gm14696
Nipsnap1	Gm19256
Oas1g	Gm24526
Olfr459	Tead1
Rnf11	Trpm3
Rpl26-ps2	
Sspo	

Supplementary Table 3:

P5-List of genes with Bmal1 Ebox regions

Enox1
Gm47200
Inhbe
Kif5c
Myo9a
Xpo5
Gm29235
Gm47030
Gm47034
Gm48841
Gm49003
Gm5161
Gm6261
Inhbc
Kif5c
Senp8
Sipa1l2
