# Supplementary material for: Neuronal Bmal1 regulates retinal angiogenesis and neovascularization in mice
Source: Commun Biol. 2022 Aug 6;5:792. doi: 10.1038/s42003-022-03774-2 (PMC9357084; doi:10.1038/s42003-022-03774-2)
Supplement: Supplementary file 3 — Reporting Summary [file 42003_2022_3774_MOESM3_ESM.pdf]

nature portfolio

Corresponding author(s): Sujata Rao, COMMSBIO-22-1115A

Last updated by author(s): Jun 20, 2022

Reporting Summary

Nature Portfolio wishes to improve the reproducibility of the work that we publish. This form provides structure for consistency and transparency in reporting. For further information on Nature Portfolio policies, see our Editorial Policies and the Editorial Policy Checklist.

Statistics

For all statistical analyses, confirm that the following items are present in the figure legend, table legend, main text, or Methods section.

n/a	Confirmed	
☐	☒	The exact sample size (*n*) for each experimental group/condition, given as a discrete number and unit of measurement
☐	☒	A statement on whether measurements were taken from distinct samples or whether the same sample was measured repeatedly
☐	☒	The statistical test(s) used AND whether they are one- or two-sided<br>*Only common tests should be described solely by name; describe more complex techniques in the Methods section.*
☐	☒	A description of all covariates tested
☐	☒	A description of any assumptions or corrections, such as tests of normality and adjustment for multiple comparisons
☐	☒	A full description of the statistical parameters including central tendency (e.g. means) or other basic estimates (e.g. regression coefficient) AND variation (e.g. standard deviation) or associated estimates of uncertainty (e.g. confidence intervals)
☒	☐	For null hypothesis testing, the test statistic (e.g. *F*, *t*, *r*) with confidence intervals, effect sizes, degrees of freedom and *P* value noted<br>*Give P values as exact values whenever suitable.*
☒	☐	For Bayesian analysis, information on the choice of priors and Markov chain Monte Carlo settings
☒	☐	For hierarchical and complex designs, identification of the appropriate level for tests and full reporting of outcomes
☒	☐	Estimates of effect sizes (e.g. Cohen's *d*, Pearson's *r*), indicating how they were calculated

Our web collection on statistics for biologists contains articles on many of the points above.

Software and code

Policy information about availability of computer code

Data collection	LASX (Leica) used for retnal falt mounts,<br>LICOR Software (Odussey) used for western blot analysis
Data analysis	Cosinor. online web based tool was used for rhythmicity anlaysis.<br>GraphPad Prism V9.0,<br>Cytoscape<br>Ingenuity Pathway Analysis (IPA)<br>Galaxy Cistrome,<br>MemeCHIP online tool,<br>Eukaryotic Promoter Database tool

For manuscripts utilizing custom algorithms or software that are central to the research but not yet described in published literature, software must be made available to editors and reviewers. We strongly encourage code deposition in a community repository (e.g. GitHub). See the Nature Portfolio guidelines for submitting code & software for further information.

Data

Policy information about availability of data

All manuscripts must include a data availability statement. This statement should provide the following information, where applicable:

- Accession codes, unique identifiers, or web links for publicly available datasets
- A description of any restrictions on data availability
- For clinical datasets or third party data, please ensure that the statement adheres to our policy

The authors declare that the (Chip-Seq) data supporting the findings of this study are available before the manuscript will be published.

Human research participants

Policy information about studies involving human research participants and Sex and Gender in Research.

Reporting on sex and gender	*Use the terms sex (biological attribute) and gender (shaped by social and cultural circumstances) carefully in order to avoid confusing both terms. Indicate if findings apply to only one sex or gender; describe whether sex and gender were considered in study design whether sex and/or gender was determined based on self-reporting or assigned and methods used. Provide in the source data disaggregated sex and gender data where this information has been collected, and consent has been obtained for sharing of individual-level data; provide overall numbers in this Reporting Summary. Please state if this information has not been collected. Report sex- and gender-based analyses where performed, justify reasons for lack of sex- and gender-based analysis.*
Population characteristics	*Describe the covariate-relevant population characteristics of the human research participants (e.g. age, genotypic information, past and current diagnosis and treatment categories). If you filled out the behavioural & social sciences study design questions and have nothing to add here, write "See above."*
Recruitment	*Describe how participants were recruited. Outline any potential self-selection bias or other biases that may be present and how these are likely to impact results.*
Ethics oversight	*Identify the organization(s) that approved the study protocol.*

Note that full information on the approval of the study protocol must also be provided in the manuscript.

Field-specific reporting

Please select the one below that is the best fit for your research. If you are not sure, read the appropriate sections before making your selection.

☒ Life sciences ☐ Behavioural & social sciences ☐ Ecological, evolutionary & environmental sciences

For a reference copy of the document with all sections, see nature.com/documents/nr-reporting-summary-flat.pdf

Life sciences study design

All studies must disclose on these points even when the disclosure is negative.

Sample size	Sample sizes were determined based on previous publications where we showed that 5 animals of control and mutants are sufficient to permit detection of differences in vascular phenotypes of 50% with a statistical power of 80% and a significance level of 0.05 (two-tailed test). For some experiments, sample sizes were determined based on mean and SD values acquired from pilot studies. In these experiments, the sample size was determine to be the optimum number of animals needed to attain a statistical significance of <P0.05 with a 90% probability.
Data exclusions	No data was excluded from the analysis
Replication	Each experiment was repeated more than 3 times. For each experiment, animals were acquired from one or more litters.
Randomization	Experiments were conducted double blind and then post analysis, genotypes were revealed. For some experiments, the conditions were known and animals were allocated into two groups, control and experimental group.
Blinding	In some experiments, where animals were maintained in constant lighting conditions, it was not possible to perform blinded analysis. However, for each experiment random animals were used from each condition and all data was compared to the control conditions.

Reporting for specific materials, systems and methods

We require information from authors about some types of materials, experimental systems and methods used in many studies. Here, indicate whether each material, system or method listed is relevant to your study. If you are not sure if a list item applies to your research, read the appropriate section before selecting a response.

Materials & experimental systems

n/a	Involved in the study
☐	☒ Antibodies
☒	☐ Eukaryotic cell lines
☒	☐ Palaeontology and archaeology
☐	☒ Animals and other organisms
☒	☐ Clinical data
☒	☐ Dual use research of concern

Methods

n/a	Involved in the study
☐	☒ ChIP-seq
☐	☒ Flow cytometry
☒	☐ MRI-based neuroimaging

Antibodies

Antibodies used	All antibodies are list are provided with vendor name and catalogue number in methods section.
Validation	*Describe the validation of each primary antibody for the species and application, noting any validation statements on the manufacturer's website, relevant citations, antibody profiles in online databases, or data provided in the manuscript.*

Animals and other research organisms

Policy information about studies involving animals; ARRIVE guidelines recommended for reporting animal research, and Sex and Gender in Research

Laboratory animals	Musculus, mixed strains and WT strains used are Bl6129/6N
Wild animals	*Provide details on animals observed in or captured in the field; report species and age where possible. Describe how animals were caught and transported and what happened to captive animals after the study (if killed, explain why and describe method; if released, say where and when) OR state that the study did not involve wild animals.*
Reporting on sex	Animals were euthanized prior to weaning and sex information was not collected. For adult animals, animals were used based on genotypes and both sexes were used in the analysis.
Field-collected samples	*For laboratory work with field-collected samples, describe all relevant parameters such as housing, maintenance, temperature, photoperiod and end-of-experiment protocol OR state that the study did not involve samples collected from the field.*
Ethics oversight	*Identify the organization(s) that approved or provided guidance on the study protocol, OR state that no ethical approval or guidance was required and explain why not.*

Note that full information on the approval of the study protocol must also be provided in the manuscript.

ChIP-seq

Data deposition

☒ Confirm that both raw and final processed data have been deposited in a public database such as GEO.

☐ Confirm that you have deposited or provided access to graph files (e.g. BED files) for the called peaks.

Data access links *May remain private before publication.*	Data will be deposited in GEO. Accession number will be provided prior to publication.
Files in database submission	P3 and P5 ChipSeq data.
Genome browser session (e.g. UCSC)	No longer applicable

Methodology

Replicates	two for each age group.
Sequencing depth	20 million
Antibodies	ab3350; Lot. no.GR297717-3, Abcam, Cambridge, MA, USA
Peak calling parameters	Sequences generated from ChIPseq experiment were filtered for quality and adapter sequences using TrimGalore! (http://www.bioinformatics.babraham.ac.uk/projects/trim_galore/). Sequences from ChIP

samples and input controls were aligned to the GRCm38 mouse reference genome (mm10) with bowtie2. Peak calling was done using
the aligned read files as input in MACS2 peak caller for broad regions of enrichment with variable-width peaks found in ChIP-Seq experiments. Peak regions were annotated using MACS2 annotation scripts and the gene list was manually curated for genes of interest.

Data quality

Describe the methods used to ensure data quality in full detail, including how many peaks are at FDR 5% and above 5-fold enrichment.

Software

Describe the software used to collect and analyze the ChIP-seq data. For custom code that has been deposited into a community repository, provide accession details.

Flow Cytometry

Plots

Confirm that:

- [x] The axis labels state the marker and fluorochrome used (e.g. CD4-FITC).
- [x] The axis scales are clearly visible. Include numbers along axes only for bottom left plot of group (a 'group' is an analysis of identical markers).
- [ ] All plots are contour plots with outliers or pseudocolor plots.
- [ ] A numerical value for number of cells or percentage (with statistics) is provided.

Methodology

Sample preparation	retinal endothelial cells isolated from the tissue.
Instrument	a Becton-Dickinson Special Order BD FACSAria II fixed alignment cuvette cell sorter, consisting of five lasers, providing 16 parameters was used for cell sorting studies.
Software	BD FACSDiva™ Software
Cell population abundance	Enriched endothelial cell populations
Gating strategy	FSC/SSC plot was used to exclude cell debris, followed by elimination of aggregates on a FSC-A/FSC-H plot. A GFP/dtTomato plot was then generated to define the subsets of interest for sorting.

- [x] Tick this box to confirm that a figure exemplifying the gating strategy is provided in the Supplementary Information.
